# Open-source lab hardware: Low noise adjustable two-stage gain transimpedance amplifier with DC offset for low-light detection

**DOI:** 10.1016/j.ohx.2021.e00233

**Published:** 2021-09-21

**Authors:** Vlad F. Cretu, Florian Kehl, Brandon C. Metz, Peter A. Willis

**Affiliations:** a NASA Jet Propulsion Laboratory, California Institute of Technology, Pasadena, CA 91109, USA; b Innovation Cluster Space and Aviation (UZH Space Hub), Air Force Center, University of Zurich, 8600 Dübendorf, Switzerland; c Institute of Anatomy, Faculty of Medicine, University of Zurich, 8057 Zurich, Switzerland; d Institute of Medical Engineering, Space Biology Group, Lucerne University of Applied Sciences and Arts, 6052, Hergiswil, Switzerland

**Keywords:** Transimpedance amplifier, Current-to-voltage converter, Photomultiplier tube, Low light detection

## Abstract

An open-source, low noise, low cost, and tunable transimpedance amplifier is presented. The compact circuit board requires few parts and costs less than $65 USD. The transimpedance amplifier is intended for low-light detection and operation with commercial photomultiplier tubes (PMTs). It provides a much more cost-effective acquisition tool compared to competitive products on the market. The system can easily be assembled and modified to suit specific current sensing applications. The amplifier features two variable gains and an adjustable DC offset to optimize dynamic range and suppress potential bias in the signal. With a target bandwidth of DC to 2 Hz and fourth-order Sallen-Key cutoff, the design is ideally suited for various applications in the field of analytical chemistry and biology, such as laser-induced fluorescence detection or chemiluminescence measurements.

## Graphical abstract

**Figure:**
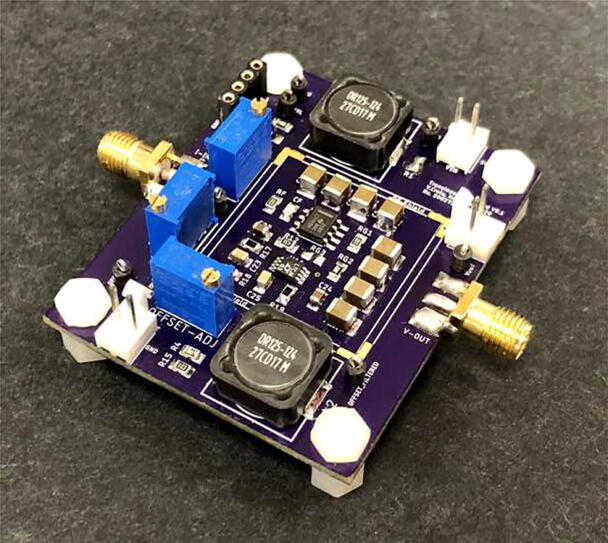

**Specifications table:** 

Hardware name	*Transimpedance Amplifier*
Subject area	• Educational Tools and Open Source Alternatives to Existing Infrastructure
Hardware type	• Measuring physical properties and in-lab sensors
Open Source License	Elsevier User License
Cost of Hardware	65 USD per board.*
Source File Repository	*https://doi.org/10.17605/OSF.IO/QUS5Y*
*The cost information contained in this document is of a budgetary and planning nature and is intended for informational purposes only. It does not constitute a commitment on the part of JPL and/or Caltech.	

## Hardware in Context

Transimpedance amplifiers (TIA) are electronic circuits to convert a current output, e.g., from a sensor, to a corresponding voltage, which can then be measured [1]. Examples of such sensors include, but are not limited to, photodiodes, photomultiplier tubes (PMTs), and capacitive micro-electromechanical systems (MEMS) such as accelerometers. TIAs are often used in combination with PMTs for low-light detection in various fields such as high-energy particle physics and astronomy, but also medicine, chemistry, or biology; for example, for analytical tools such as spectrophotometry, (laser-induced) fluorescence detection, or chemiluminescence [2], [3], [4], [5], [6], [7], [8]. A multitude of TIA architectures, such as Common Gate, Integrator-Differentiator, Capacitive or Resistive Feedback, or Cascode topologies, are well known and described in detail in the literature [9]. A comprehensive theoretical discussion and comparison of the different topologies are beyond the scope of this hardware-focused manuscript, and the interested reader is referred to the abovementioned literature.

During the development of the presented hardware, the authors realized that although there is a lot of textbook knowledge and design principles available for TIAs, only relatively costly commercial TIAs are available on the market. Examples include, but are not limited to: AMP1XX series (Price range: USD 275 – 490), TIA60 (USD 1048), PDA200C (USD 942), all from Thorlabs (Thorlabs, USA), various products from Hamamatsu (Hamamatsu Photonics K.K., Japan), such as products C6438, C7319, or C999, or evaluation boards from Analog Devices Inc., USA (ADA4530-1R-EBZ-TIA, USD 639), Maxim Integrated, USA (MAX40660EVKIT, USD 127), or Texas Instruments, USA (OPA857EVM, USD 128). Hence, an open-hardware, customizable low-cost alternative TIA is presented, equipped with variable gain and offset, targeted for the development of scientific instrumentation, sensor, and low-light detectors, which is easy to use for non-experts. This TIA is equipped with an internal low-pass filter (LPF) and has been designed and demonstrated here for use with commercial PMTs. Additionally, it can directly power an external PMT via a low-pass filtered supply and tune the PMT gain via a control voltage provided by the PMT. The design of the TIA’s LPF was optimized for noise rejection and low-light detection for relatively slow signals (<10 Hz) with a potentially high background or dark current, which then also allows for longer integration times of the acquired signal.

## Hardware Description

Our board offers the following features:

• Easy benchtop testing
• Low cost
• Compatibility with standard commercial photomultiplier tubes (PMT) or photodiodes
• Adjustable transimpedance amplifier gain: 224 kV/A – 784 kV/A
• Adjustable gain settings for external PMTs
• Low bandwidth: DC – 2 Hz
• High precision, ultra-low noise operation
• Adjustable baseline DC offset (to match the dynamic range of the data acquisition system)

**Fig. 1:**
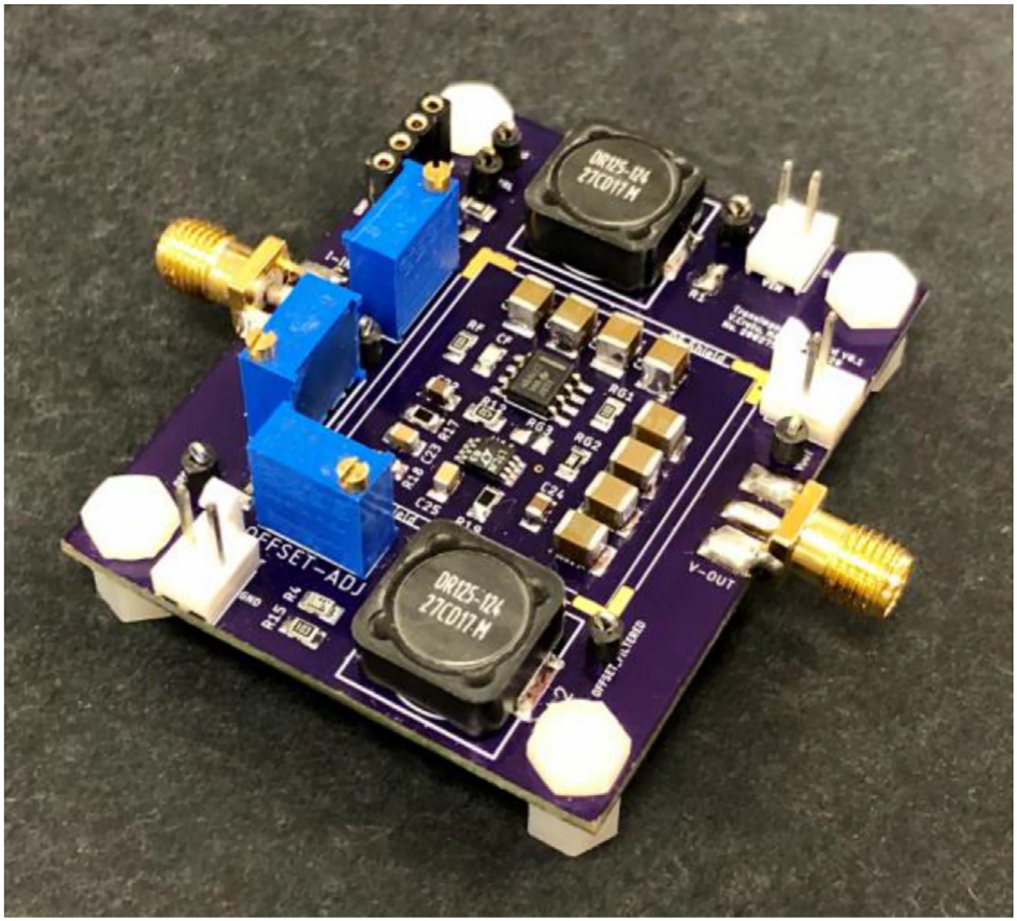
Photograph of the assembled transimpedance amplifier. For visibility purposes, the radio frequency shield has not been soldered to the board in this picture.

The printed circuit board (PCB, Fig. 1) was designed using Autodesk EAGLE (Autodesk, CA, USA). This is a two-layer board that was fabricated with OSH Park, OR, USA, but can be manufactured with any other suitable board house.

**Fig. 2:**
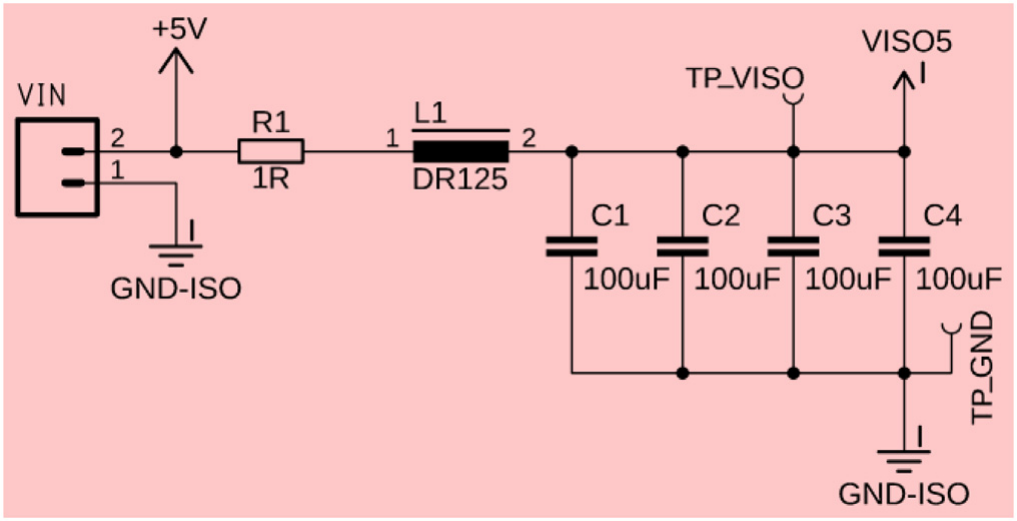
5 V are applied to VIN to power the transimpedance amplifier and is passed through a 200 Hz cutoff LPF, comprised of one resistor R1, an inductor coil L1, and a series of parallel capacitors, C1 to C4. There are two test points (TP), TP_VISO, and TP_GND, to conveniently measure the noise-isolated voltage, V_ISO,_ and GND.

To power the board, an input voltage of 4.5 to 5.5 V is required. However, a stable 5 V source is recommended for optimal performance. The board has an internal passive LPF with a 200 Hz cutoff to reject unwanted higher frequency noise from the power supply and ensure a stable supply voltage for the amplifier. Fig. 2 shows the schematics of the voltage input low-pass filter.

**Fig. 3:**
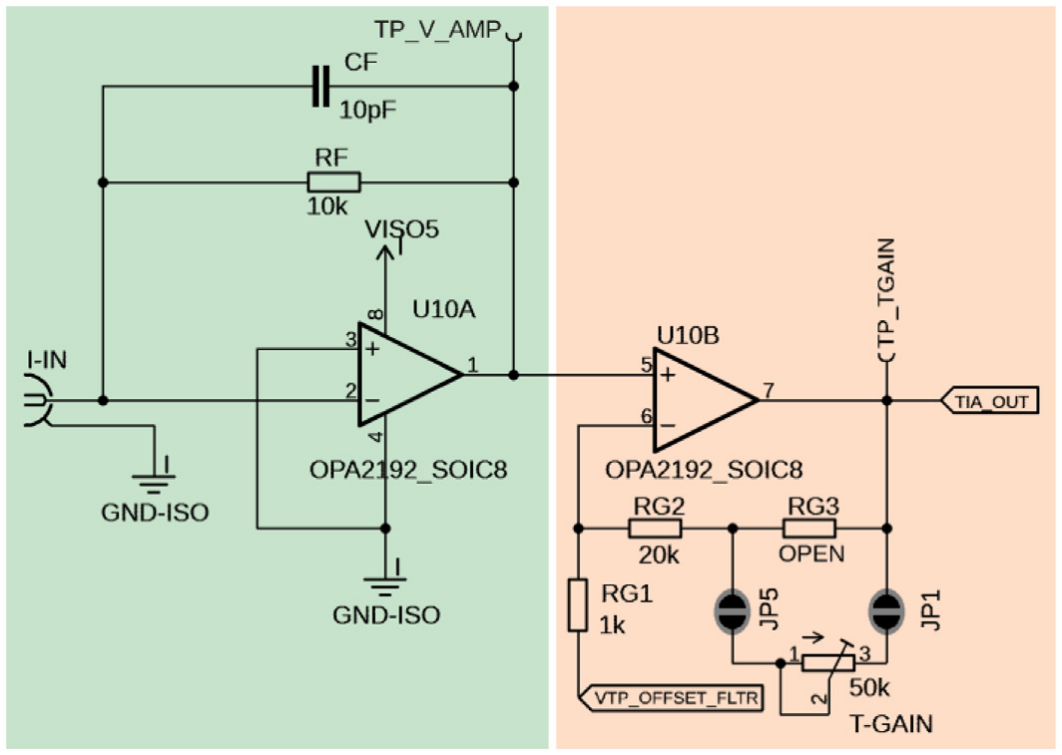
Transimpedance amplifier. Left (green): The current signal is connected to I-IN and converted to a voltage by the transimpedance amplifier with a static gain of 10 k. Feedback resistor R_F_ can be replaced with other values to change the fixed gain, while the capacitor C_F_ can be changed to adjust the response time. Right (orange): Adjustable DC offset correction with adjustable 2nd stage signal amplifier. When solder jumpers JP1 and JP5 are bridged, the second stage gain can be set with the T-Gain potentiometer, while RG3 is open. If the gain doesn’t have to be adjustable, JP1 and JP5 can be left open, and a fixed value resistor can be soldered in position RG3 instead. (For interpretation of the references to colour in this figure legend, the reader is referred to the web version of this article.)

At its core, the TIA board consists of the transimpedance amplifier itself (Fig. 3) and a filter stage (Fig. 5). The filter stage will be explained in more detail below. The transimpedance amplifier operates with an OPA2192, from Texas Instruments, TX, USA, which is an operational amplifier (op-amp) for low noise and rail-to-rail operation (Fig. 3). The OPA2192 was chosen for the first stage of the amplifier, the current-to-voltage conversion for its low noise ($5.5nV/\sqrt{\mathrm{Hz}}$) and stability.

Due to the input voltage offset of the OPA2192, the input impedance is approximately 1-ohm across its entire range 1 μA–20 μA. This is confirmed by our LTSpice simulation (Fig. 10). The transimpedance amplifier is split into two different gain stages, one fixed gain set by resistor R_F_, and a variable transimpedance gain (T-Gain). The T-Gain gain can be adjusted using the T-Gain potentiometer and is adjustable by leaving RG3 open and connecting solder jumpers JP1 and JP5. The following equations show the behavior of adjusting resistors and potentiometers on the TIA board. Beginning with the transimpedance stage, the amplifier has a set gain of 10 k, which is denoted by the following equation:

(1) $${\text{V}}_{\text{TP}\_\text{V}-\text{AMP}}={\text{I}}_{\text{in}}*\;\left({\text{R}}_{\text{RF}}\right).$$

where V_TP_V-AMP_ is the output voltage of the first stage in volts (V), I_in_ the input current (in A), and R_RF_ the resistance of resistor R_F_ in Ohms (Ω). By swapping resistor R_F_ with a different resistor value, the user can adjust the first stage gain accordingly.

In a TIA amplifier design, C_CF_, or feedback capacitor, is crucial for stability. C_CF_ dictates transient response, or the rise and fall of the output signal, as well as the steady-state output. It is important to choose an optimal C_CF_ value to improve response time, reduce overshoot, and eliminate oscillation. In this design, however, the transient response is dictated by the two-stage filter in Fig. 5 rather than the C_CF_. C_CF_ is still important in ensuring system stability, and the authors recommend a small value of around 10 pF.

The second amplification stage is adjustable and can be tuned to the desired sensitivity. By adjusting the T-Gain (“transimpedance gain”) potentiometer, the gain can be increased or decreased. Eq. (2) below calculates the gain based on the resistance set by the T-Gain potentiometer. If the user decides that this gain needs to be higher than the potentiometer can offer, resistor RG2 can be exchanged for a higher resistance value, thus increasing the gain. One way to reduce noise and create a fixed and reliable gain is to desolder the solder jumpers JP1 and JP5, and replacing resistor RG3 with the desired, fixed resistance. To further reduce potential electromagnetic interference (EMI), a radio frequency shield can be soldered around the central portion of the amplifier stage.

(2) $$V_{TP\_T-GAIN}=\;\left(V_{TP\_V-AMP}-\;V_{TP\_OFFSET\_FLTR}\right)*\left(\frac{R_{RG2}+R_{RG3}}{R_{RG1}}+1\right).$$

V_TP_T-GAIN_ is the TIA_OUT voltage at the output of the TIA stage, which can be measured at test point TP_TGAIN, and V_TP_V-AMP_ is the pre-amplified signal in Eq. (1). The gain can be set with fixed resistor values RG1 through RG3, or resistor RG3 can be left open and varied by the T-GAIN potentiometer.

**Fig. 4:**
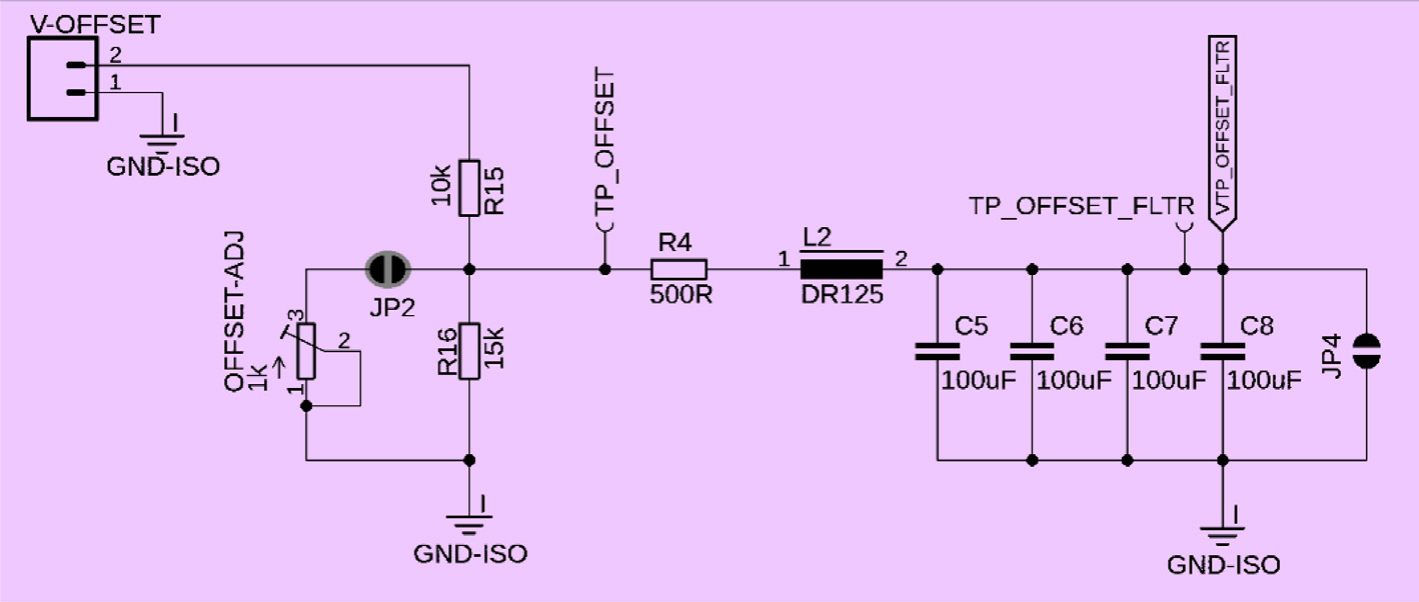
Circuit to adjust offset: a 200 Hz cutoff passive LPF created by a series of parallel capacitors, C5 to C8, a resistor R4, and an inductor coil L2. When 5 V is applied to V-offset, the voltage divider, comprised of resistor R15 and Offset-Adj potentiometer, allows for a maximum DC offset of the signal range between 0 and 3 V. JP2 should be closed when DC Offset-Adj needs to be customized; otherwise, a 3 V DC offset is imposed on the signal. JP4 should be closed when a DC offset is not desired. If JP4 is closed, there should not be a voltage applied at the V-offset connector. A closed dot around a solder jumper (e.g., JP2) indicates that by default, this jumper should be closed, while the symbol for JP4 marks an open jumper.

An additional feature of this transimpedance amplifier is that a variable DC offset (measured at TP_OFFSET_FLTR) can be subtracted from the pre-amplified V_TP_V-AMP_ signal before it’s amplified in the second stage. This enables shifting the linear range of the sensor and removing potential background signals. By doing so, signal saturation can be avoided and optimized to meet the dynamic range of the input source, such as a PMT, while maintaining high sensitivity. This ultimately results in a higher signal-to-noise ratio (SNR). The DC offset can be adjusted by turning the Offset-Adj trimmer (Fig. 4). The DC voltage offset can be calculated using Eq. (3), which accounts for JP2 being connected. If JP2 is not connected, R_p_ can be substituted with R16 directly. JP2 can be opened if the desired offset for a particular application is reached. In this case, an equivalent resistor with the corresponding resistor value has to be installed at R16. This makes the system more reliable and robust by removing any noise picked up by the potentiometer.

(3) $$V_{TP_{\mathrm{OFFSET}}}=V_{\mathrm{offset}}*\left(\frac{R_{p}}{R_{R15}+R_{p}}\right),where\;R_{p}=\left(\frac{R_{OFFSET\_ADJ}*R_{R16}}{R_{OFFSET\_ADJ}+R_{R16}}\right),$$

**Fig. 5:**
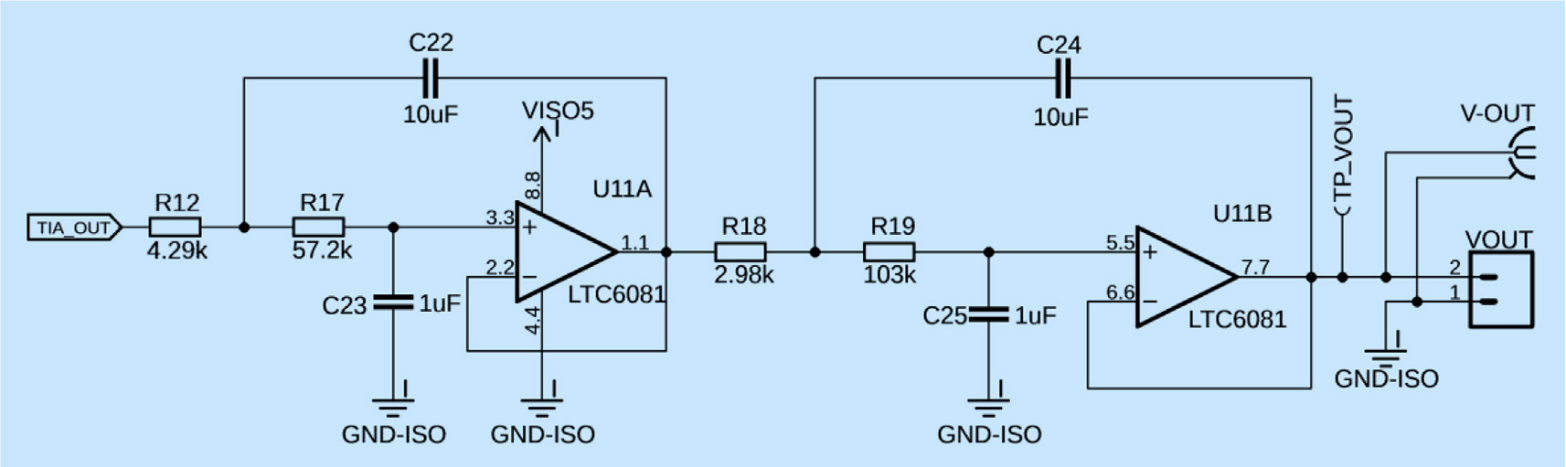
Two-stage, Sallen-Key topography, fourth-order Bessell filter with a 2 Hz cutoff. The output from the TIA (Fig. 3) is cleaned up by the LPF depicted in this figure. The conditioned signal can then be acquired at V-OUT.

The 2nd component stage of the Transimpedance amplifier is coupled to an active low pass filter, shown in Fig. 5. To achieve this low noise performance, we have chosen LTC6081 from Analog Devices, MA, USA, which is also a rail-to-rail op-amp for ultra-low-noise applications. In this design, it is ideally suited for a high-precision second stage, fourth-order Bessel filter. The filter has a 2 Hz cutoff (−3 dB) and 10 Hz (−40 dB) stopband. For inexperienced users, it is not recommended to change capacitors and resistors on the LTC6081 filter stages to adjust low pass cutoff and stopband frequencies. This is to prevent output ripple of the signal. Additionally, it is important to choose closely matched resistors and capacitors with low tolerance to ensure low noise operation and up to specification filtering

**Fig. 6:**
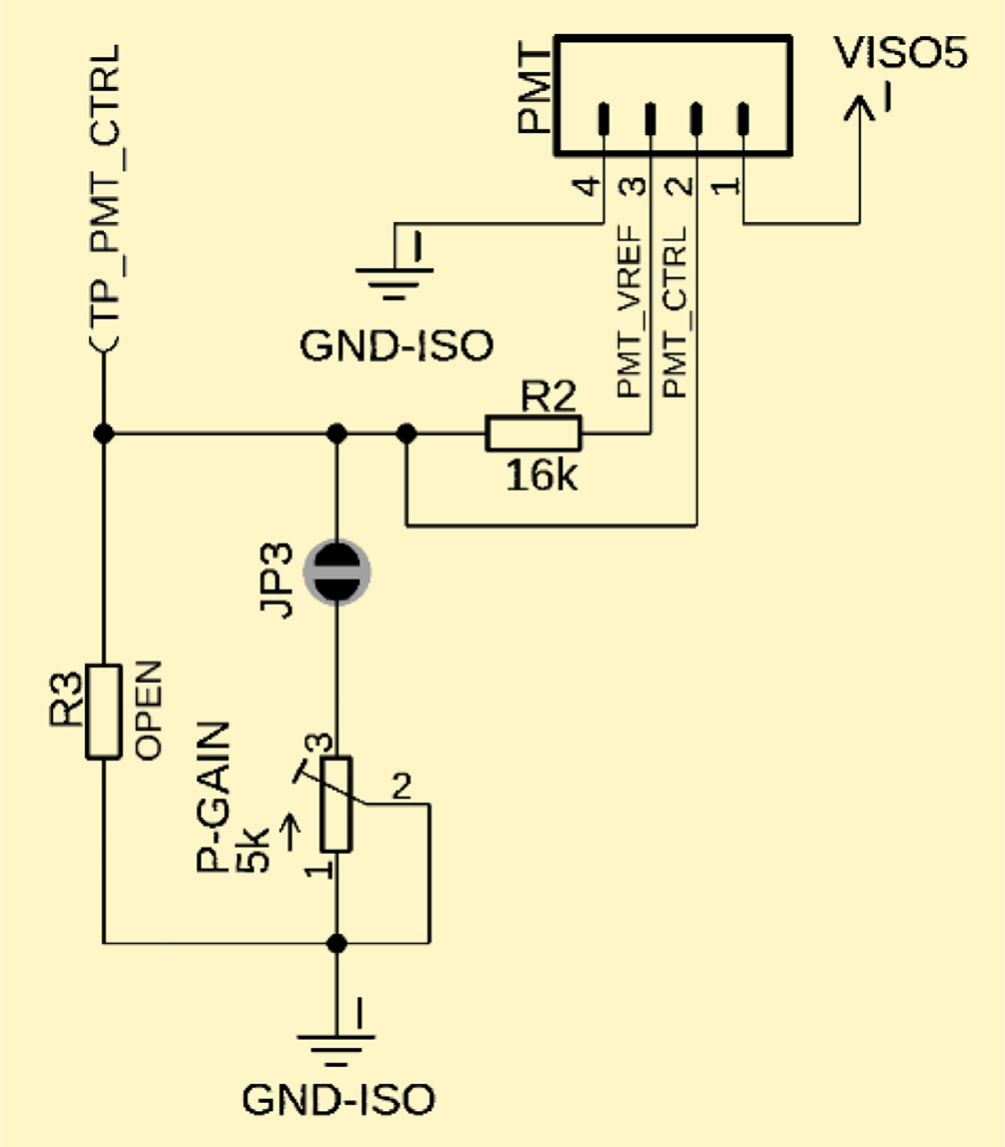
Interface to an external PMT: Pin 1 and 4 supply 5 V power and ground. Some PMTs are equipped with an internal voltage reference and a control input for its internal gain. These can be connected to pin 3 and 2, respectively. The PMT gain can be set with the P-gain potentiometer or R3.

The presented TIA board is additionally equipped with a connector to power and control an external PMT (Fig. 6). Pins 1 and 4 provide 5 V power and ground to the PMT, respectively. Many commercial PMTs provide an internal reference voltage as a stable source for the control voltage. This reference voltage is connected to Pin 3 of the PMT header connector and via a voltage divider, defining the control voltage at Pin 2 to bias the PMT’s sensitivity. Contrary to the fixed gain set by R_F_ and the T-Gain, adjusting the P-Gain directly controls the gain of the PMT and adjusts how much current it outputs based on its light input. The set PMT control voltage can be tuned by the P-Gain potentiometer and measured at test point TP_PMT_CTRL. If a fixed P-gain is desired, JP3 can be opened, and a resistor with the desired value can be soldered in place of R3.

**Fig. 7:**
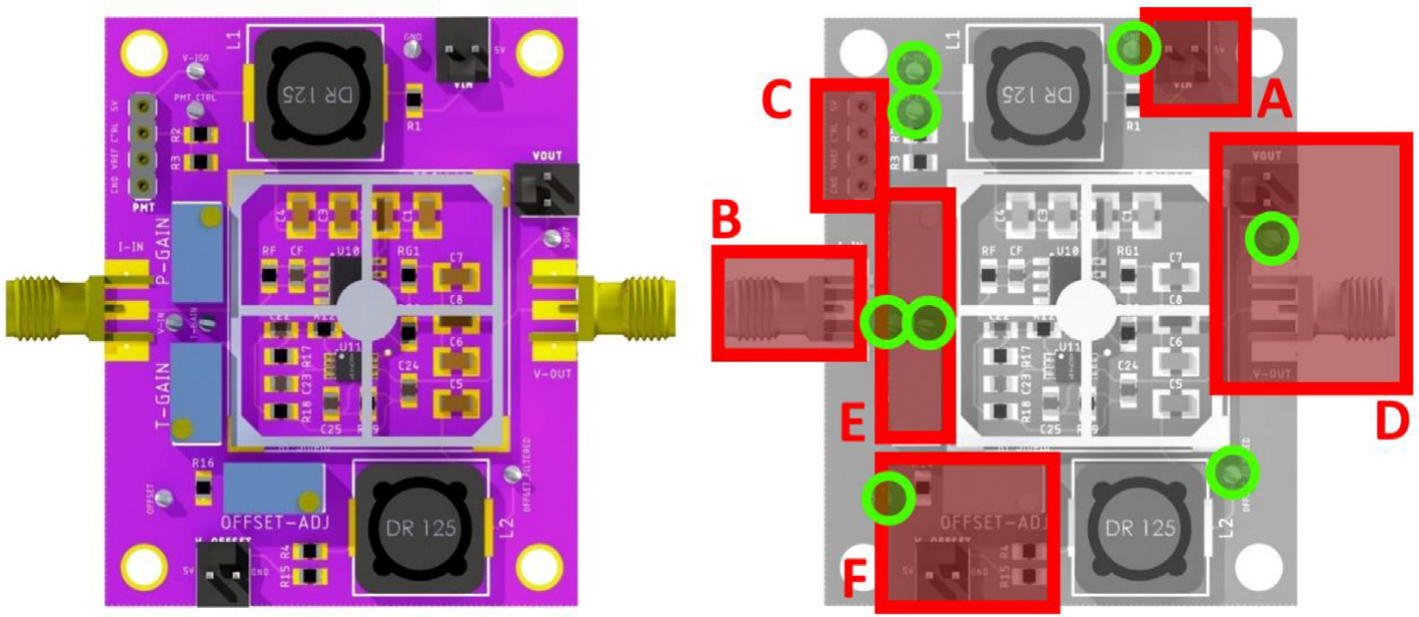
CAD top view of the assembled board (left), together with a map of the board, with A) 5 V input, B) current input, C) PMT power and control, D) signal output, E) potentiometers to adjust P and T-Gain, F) input, and potentiometer for offset control. Green circles highlight the test points where various signals can be probed. (For interpretation of the references to colour in this figure legend, the reader is referred to the web version of this article.)

Fig. 7 provides a top view of the board and highlights the various elements, including where they are physically located on the board. The solder jumpers are on the backside of the PCB.

**Fig. 8:**
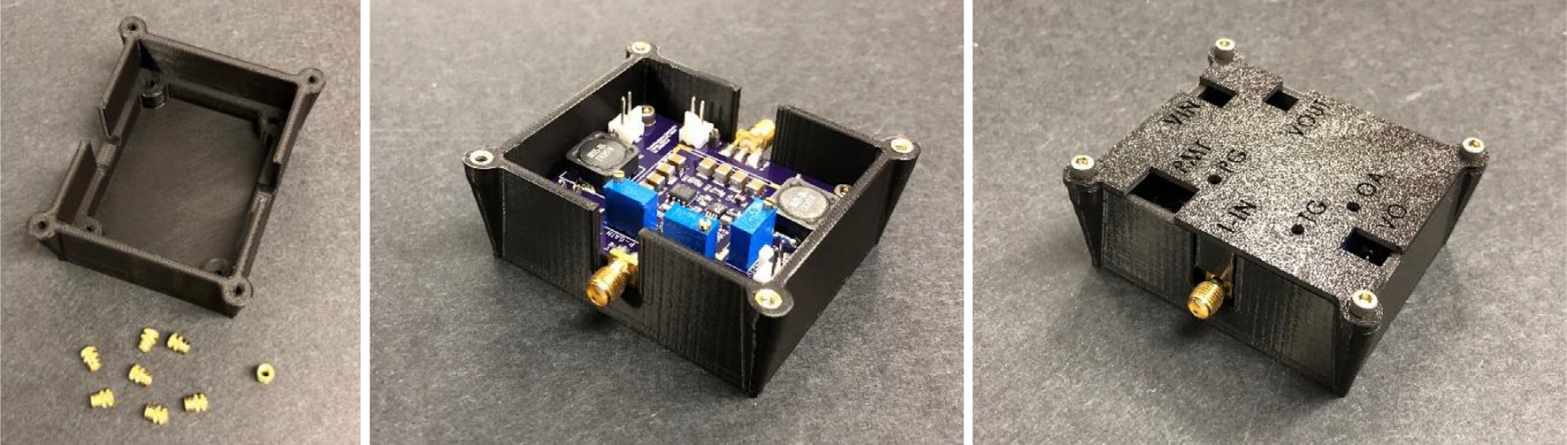
Left: 3D printed enclosure bottom with heat inserts. Middle: Installed TIA in enclosure. Right: TIA enclosure with lid and access holes for connectors and potentiometers.

If desired, a CAD file for a 3D printable, custom enclosure for the TIA electronics is provided with this manuscript, comprising of a bottom part and a lid (Fig. 8).

## Design Files

The design files consist of the documents needed to order and assemble the board. It includes the following files:

• Schematic, a .sch file
• Layout, a .brd file
• Enclosure bottom, a .step file
• Enclosure lid, a .step file

**Design files summary:** 

Design file name	File type	Open source license	Location of the file
TIA Board.sch	Schematics	*Elsevier User License*	*https://osf.io/qus5y/*
TIA Board.brd	Layout	*Elsevier User License*	*https://osf.io/qus5y/*
TIA Board.lbr	Library	*Elsevier User License*	*https://osf.io/qus5y/*
TIA Parasolid.x_t	CAD	*Elsevier User License*	*https://osf.io/qus5y/*
TIA Box.STL	CAD, 3D-printing	*Elsevier User License*	*https://osf.io/qus5y/*
TIA Lid.STL	CAD, 3D-printing	*Elsevier User License*	*https://osf.io/qus5y/*

**Table:** 

File	Description
TIA Board.sch	Autodesk Eagle schematic file. The PCB schematic can be viewed and edited if desired.
TIA Board.brd	Autodesk Eagle board file. Needed to produce the PCB.
TIA Board.lbr	Autodesk Eagle part library for the PCB.
TIA Parasolid.x_t	3D model of the PCB assembly.
TIA Box.STL	STL-File to 3D-print the enclosure box.
TIA Lid.STL	STL-File to 3D-print the enclosure lid.

## Bill of Materials

The part selection only requires that the transimpedance and filter capacitors and resistors be as close to the design as possible, with the lowest tolerance possible. This is to ensure the lowest noise capability and up-to-spec performance.

Please note that prices may change

**TIA board:** 

Designator	Component	Number	Cost per unit – USD	Total cost – USD	Source: Digikey part number	Material type
C1, C2, C3, C4, C5, C6, C7, C8	CAP CER 100UF 16V X5R 1210	8	2.07	24.84	587-3152-1-ND	Ceramic
TP1, TP2, TP3, TP4, TP5, TP7, TP8	PC Test point	8	0.4	3.2	36-5001-ND	
V-OFFSET, VIN, VOUT	CONN HEADER VERT 2POS 2.54	3	0.17	0.51	900-0022232021-ND	
R15, R_F_	10K RESISTOR 0.1% 1/8W 0805	2	0.33	0.66	P10KDACT-ND	Ceramic
C22, C24	CAP CER 10UF 10V X7R 0805	2	0.83	1.66	399-15693-1-ND	Ceramic
C23, C25	CAP CER 1UF 50V X7R 0805	2	0.38	0.76	311-3498-1-ND	Ceramic
L1, L2	FIXED IND 1MH 570MA 1.69 OHM	2	1.23	2.46	513-1745-1-ND	
I-IN, V-OUT	CONN SMA JACK STR EDGE MNT	2	1.7	3.4	CON-SMA-EDGE-S-ND	
P-GAIN, T-GAIN	TRIMMER 50K OHM 0.5W PC PIN TOP	2	2.41	4.82	3296Y-503LF-ND	
R1, R2	RES SMD 1 OHM 5% 1/2W 0805	2	0.24	0.48	P19220CT-ND	Ceramic
C_CF_	CAP CER 10PF 50V C0G/NP0 0805	1	0.1	0.1	1276-2561-1-ND	Ceramic
R4	RES SMD 499 OHM 0.5% 1/5W 0805	1	0.41	0.41	MCU0805-499-MDCT-ND	Ceramic
PMT	CONN HEADER VERT 4POS 2.54MM	1	0.28	0.28	WM4202-ND	
R19	RES 102K OHM 0.1% 1/8W 0805	1	0.33	0.33	P102KDACT-ND	Ceramic
R16	RES 15K OHM 1% 1/8W 0805	1	0.1	0.1	A129763CT-ND	Ceramic
RG1	RES 1K OHM 0.1% 1/8W 0805	1	0.33	0.33	P1.0KDACT-ND	Ceramic
OFFSET-ADJ	TRIMMER 1K OHM 0.5W PC PIN TOP	1	2.41	2.41	3296Y-102LF-ND	
R18	RES 3K OHM 0.1% 1/8W 0805	1	0.33	0.33	P3.0KDACT-ND	Ceramic
RG2	RES SMD 20K OHM 1% 1/8W 0805	1	0.1	0.1	311-20.0KCRCT-ND	Ceramic
R12	RES 4.3K OHM 0.1% 1/8W 0805	1	0.33	0.33	P4.3KDACT-ND	Ceramic
R17	RES 57.6K OHM 0.1% 1/8W 0805	1	0.33	0.33	P57.6KDACT-ND	Ceramic
R3	RES SMD 5K OHM 1% 1/8W 0805	1	0.14	0.14	541-4321-1-ND	Ceramic
U11	IC OPAMP GP 2 CIRCUIT 8MSOP	1	4.49	4.49	LTC6081CMS8#TRPBFCT-ND	
U10	OPA2192IDR IC OPAMP GP 2 CIRCUIT 8SOIC	1	4.08	4.08	296-42106-6-ND	
RF-S (bottom)	RF SHIELD CAGE 1.058″ X 1.058″ SMD	1	2.01	2.01	1798-1181-1-ND	
RF-S (top)	RF SHIELD CAP 1.034″ X 1.034″ SMD CAP	1	0.48	0.48	1789-1182-ND

**Enclosure:** 

Designator	Component	Number	Cost per unit – USD	Total cost – USD	Source: McMaster-Carr part number	Material type
Heat Inserts	4-40 Tapered Heat-Set Inserts for Plastic	8	0.13	1.04	93365A122	Brass
Screws	18-8 Stainless Steel Socket Head Screws	8	0.05	0.40	92196A105	Stainless Steel

## Build Instructions

TIA Board:

• Populate the printed circuit board (PCB) using a hot air solder iron or reflow oven for surface-mount device (SMD) components. Soldering the SMD op-amps and 0805 components before moving forward with the larger SMD and through-hole components is recommended.
• For default operation (use of potentiometers to adjust gains and offset), close solder jumpers JP1, JP2, JP3, and JP5, and leave JP4 open. Unless a fixed resistor is desired, do not install a resistor for R3 and RG3.
• Solder the through-hole components using a solder iron and solder.
• If desired, the radio frequency shield can be installed to reduce any external EMI.
• Install board spacers or standoffs at the four corners of the PCB to avoid any unwanted short-circuit with a potentially conductive surface, or use the provided 3D printable enclosure.

Enclosure:

• 3D print enclosure bottom and lid.
• Install 4-40 (or M3) heat inserts or tap screw holes directly.
• Mount TIA board inside the bottom enclosure using four 4-40 (or M3) screws.
• Close lid using four 4-40 (or M3) screws.

## Operation Instructions

The following will explain all user-adjustable parameters to allow the transimpedance to be optimally used for any specific situation.

• Connect the input signal (e.g., from a PMT) to the I_IN SMA connector. The default input current should conform to the current limits (max. 5 μA for 784 kV/A, or max. 19 μA for 224 kV/A), depending on the gain settings. For example, if the gain is set to maximum (784 kV/A), the resulting current would start to saturate at 5 μA. Hence, the maximum input current would be anything below that. Similarly, if the gain is set to 224 kV/A, the maximum detectable current would be 19 μA. If a wider current range is desired, the first gain stage can be adjusted by replacing resistor R_F_, as previously explained.
• If applicable, connect the PMT control lines to the female header pins consisting of 5 V, GND, PMT_Vref, and PMT_Ctrl.
• Connect the Vout SMA connector to an oscilloscope or the header pins of an analog-to-digital converter (ADC) for signal acquisition.
• Apply 5 V to the VIN using a stable low noise source. The cleaner the source, the better the performance of the transimpedance amplifier.
• Measure the voltage at TP_PMT_CTRL to be in accordance with the specific PMT gain range. The voltage can be adjusted by closing solder jumper JP3 and rotating the P-Gain 50kΩ potentiometer to achieve the desired PMT gain. The PMT gain can be adjusted during operation as well. For a more stable, low-noise operation, the potentiometer can be replaced with a fixed equal resistance. To do this, desolder JP3, and solder the resistor with the corresponding value in R3.
• The secondary gain adjustment is the transimpedance amplifier gain. This is set by first bridging solder jumpers JP1 and JP5, then rotating the T-Gain 50 kΩ potentiometer to achieve the desired output gain by monitoring the voltage at TP_Gain. Similar to the P-Gain potentiometer, to achieve higher output stability and reduced noise, JP1 and JP5 can be desoldered, while RG3 can be soldered with a fixed equal resistance to the T-Gain potentiometer.
• If a DC offset is not desired, JP4 should be soldered. This provides a cleaner signal as the Offset-adj potentiometer might induce noise or signal drift. In this case, the connector V-offset should be left floating.
• If the DC offset capability is desired, first desolder JP4. Next, a max. 5 V potential should be applied at the V-Offset connector. JP2 should be soldered to allow the Offset-adj 1kΩ potentiometer to act as a voltage divider, with a range from 3 V down to 455 mV. The voltage at V_TP_OFFSET_FLTR_ will be subtracted from the signal before the T-Gain potentiometer stage. Turning the Offset-adj potentiometer will shift the DC signal. If a particular linear range is found to be optimal for the measured signal, the stability and noise can be optimized by desoldering JP2 and replacing resistor R16 with an equivalent combined parallel resistance formed between Offset-adj potentiometer and R16.

**Key TIA Specs:** Validation and characterization

Transimpedance Gain	Bandwidth	Rise/Fall Time (10–90%)	input current limit	Mass (weight)
224 kV/A – 784 kV/A	DC – 2 Hz	∼200 ms rise and 177 ms fall time	5.5 μA max. for 784 kV/A and 19 uA max. for 224 kV/A	24.7 g

**Fig. 9:**
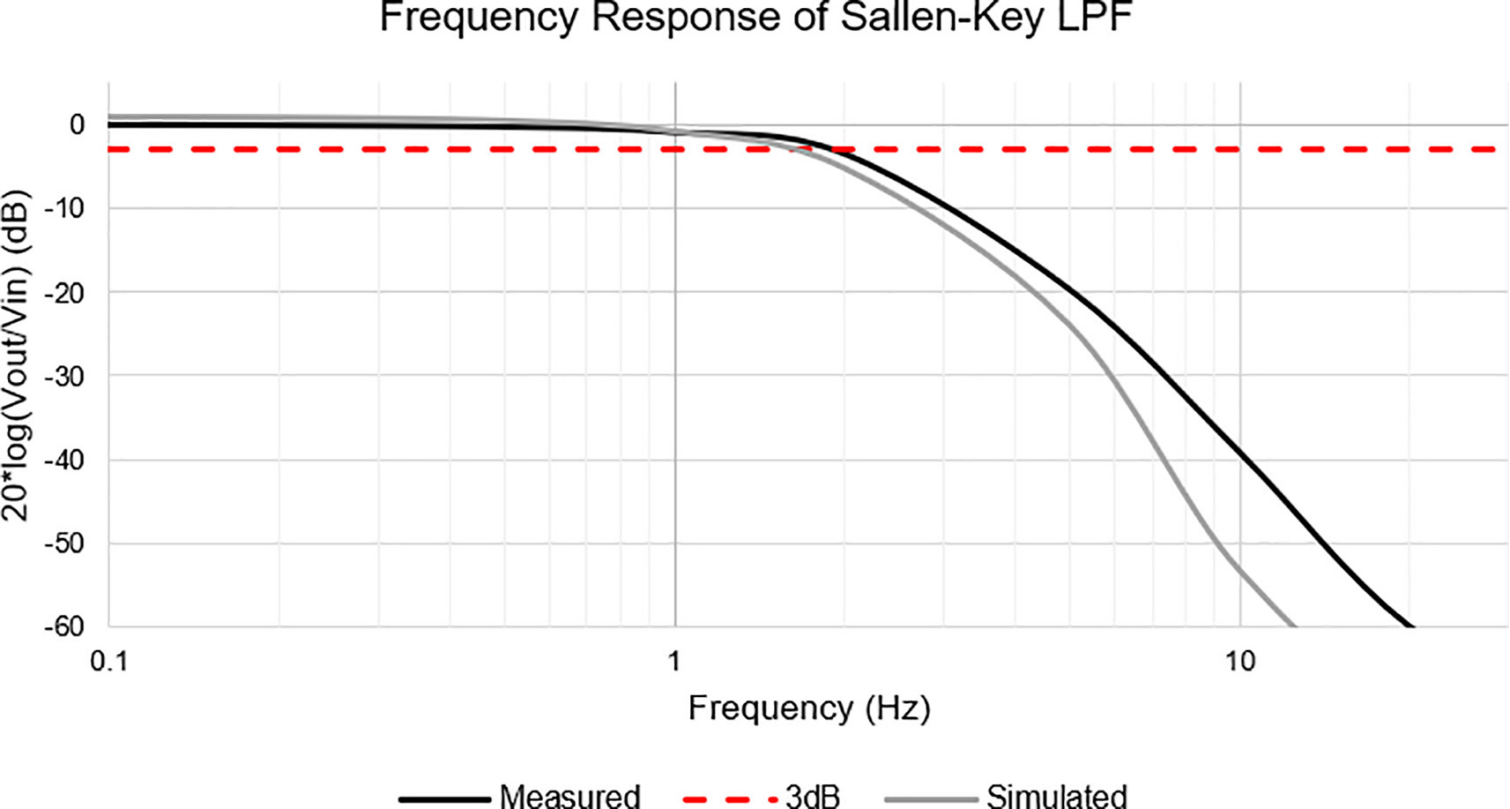
Simulated vs. Measured Frequency Response of the LPF. The red line represents −3 dB cutoff frequency. (For interpretation of the references to colour in this figure legend, the reader is referred to the web version of this article.)

The frequency response of the TIA is dominated by the two-stage Sallen-Key Filter. This two-stage filter circuit was designed using the parameters of the LTC6081 with 1% tolerance resistors and 10% tolerance capacitors. The input to the filter was an AC sinusoidal signal with a fixed amplitude. The frequency of this AC source was swept between 0.1 Hz and 100 Hz. The measured frequency response was also similarly captured, applying an AC sinusoidal source of 5 V peak to peak. The frequency was also cycled for values between 0.1 Hz and 100 Hz, and the output peak to peak voltage was recorded with an oscilloscope (HMO1024, Rohde & Schwarz, Germany). The resulting magnitude graph (in dB) of both measurements is plotted in Fig. 9.

**Fig. 10:**
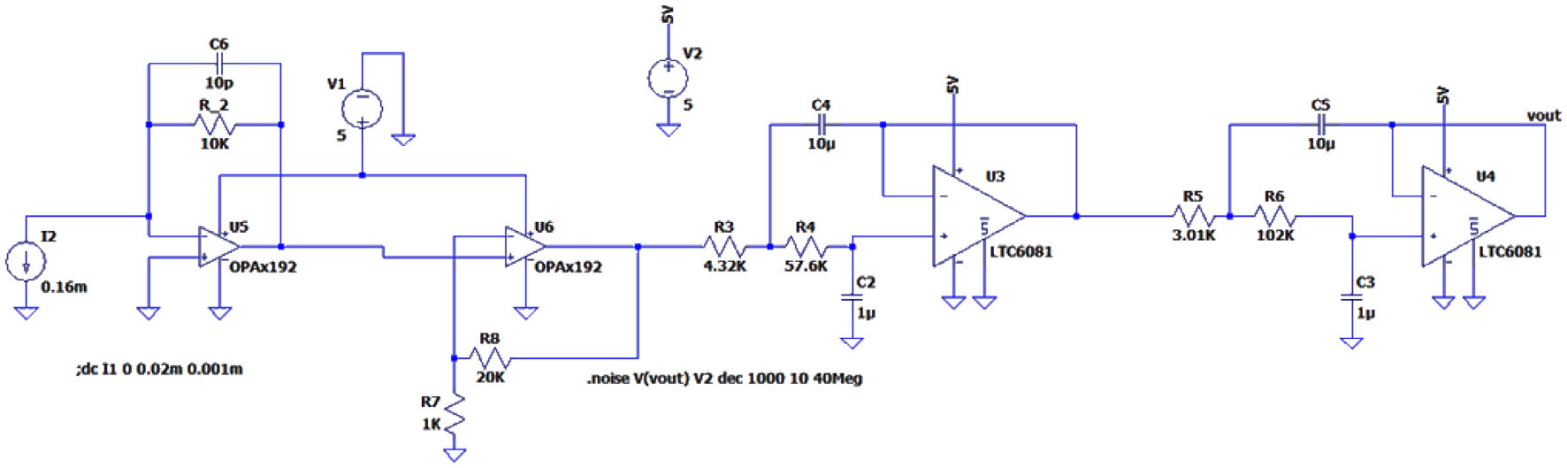
Frequency analysis circuit without potentiometers.

**Fig. 11:**
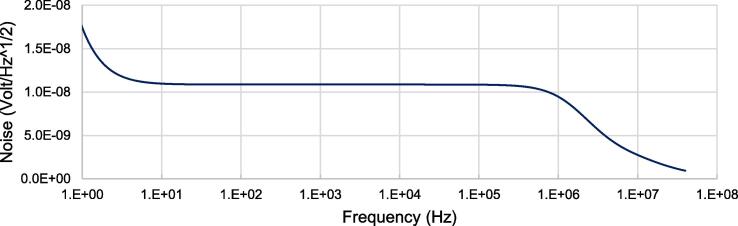
Simulated frequency analysis sweep from 1 Hz to 10 MHz.

LTSpice was used to simulate the total noise analysis of the system. To ensure the lowest noise performance, the potentiometers have been removed for this simulation. We recommend using fixed resistors to achieve similar performance. The frequency was then swept from 1 Hz to 40 MHz. The test circuit and resulting noise analysis are shown in Fig. 10, Fig. 11, respectively.

The presented TIA has been tested, and its performance verified using a commercial PMT (from Hamamatsu, Japan, model: H10721-210). The PMT’s signal output was connected to the TIA’s input (I-IN), the control wires to the PMT connector (Fig. 6) to provide power, ground, and a PMT gain control voltage (P-Gain). Power (5 V) was provided to both the board to VIN as well as the Offset Input by a standard laboratory power supply. The TIA’s output was acquired with an oscilloscope (HMO1024, Rohde & Schwarz, Germany) at 5 kHz and averaged over 1.2 s.

**Fig. 12:**
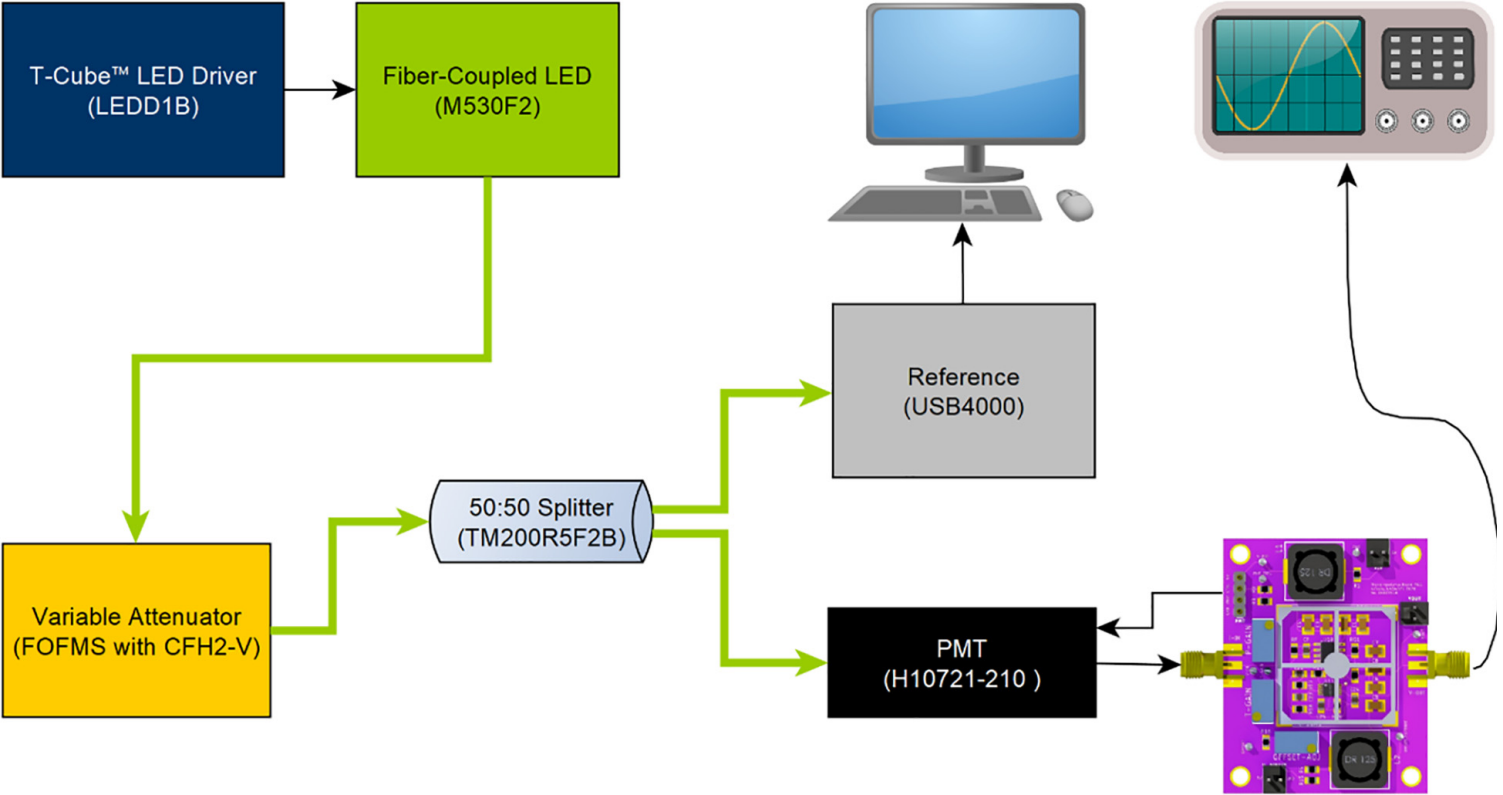
Test setup: An LED driver was used to vary the brightness of a fiber-coupled LED. The signal was attenuated before sending it into a 50:50 fiber splitter. One of the splitter’s arm was connected to a spectrometer as a calibrated reference, the other arm to a PMT. The PMT was powered and controlled by the TIA board. The TIA converted and amplified the PMT’s current output to a voltage, which was then acquired by an oscilloscope.

As a light source, a fiber-coupled LED at 530 nm (M530F2, Thorlabs, USA), connected via 0.22NA, 50 um core multi-mode fiber patch cables (M16L01, Thorlabs), to an in-line fiber-coupled variable attenuator (FOFMS with CFH2-V, both Thorlabs) was used (Fig. 12). The LED brightness was manually controlled using a T-Cube™ LED Driver (LEDD1B, Thorlabs). The attenuated signal was then split by a 50:50 multi-mode fiber optic coupler (TM200R5F2B, Thorlabs), while one arm acted as a reference beam, and the other was connected to the abovementioned H10721-210 PMT via an FC/PC interface connector (E5776, Hamamatsu, Japan). The electrical connections of the PMT were then connected to the TIA. An Ocean Optics USB4000 spectrometer (Ocean Insight Inc., USA) was connected to the reference arm, and the light intensity over the entire emission spectrum of the LED was recorded with Ocean Insight’s SpectraSuite Software. To express the measurements in absolute units, the optical reference signal was first calibrated using a USB photon counting head (H11890-210, Hamamatsu, Japan), with an integration time of 10 ms. The USB photon counting head was not used during the measurements as a direct comparison, as it started to saturate around 200,000 photons per 10 ms or 2*10^7^ photons per second, and has a fixed gain. Since the goal was to demonstrate not only variable gain but also an increased, tuneable linear range of the presented TIA compared to the USB PMT, the aforementioned spectrometer with a wide measurement range was used as our reference signal.

To demonstrate how the TIA works and how it can be used for different applications, a series of measurements were performed with the above mentioned setup. To highlight the effect of each parameter individually, PMT gain, transimpedance gain, and offset adjust, one of the parameters was varied while the other two were kept constant. The TIA response was then recorded with the abovementioned setup at different light intensities and for three different settings per parameter.

**Fig. 13:**
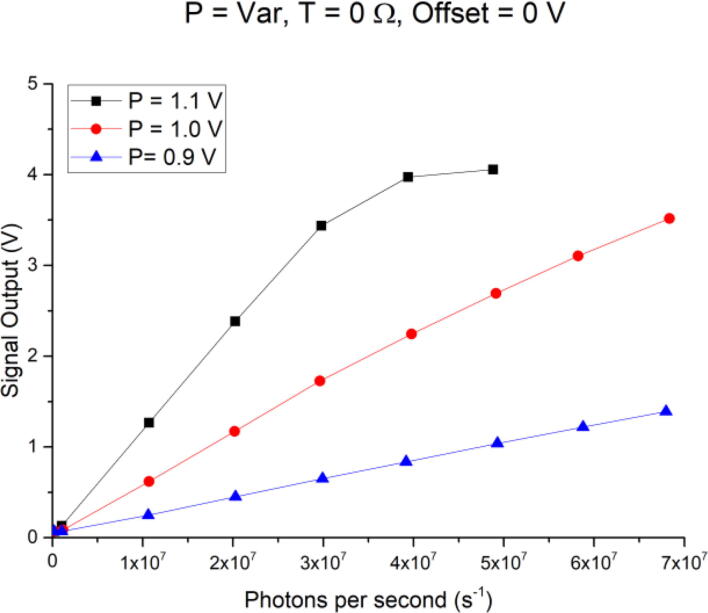
Output signal for varying P-gain settings (0.9 V, 1 V, 1.1 V), with a fixed resistance for the T-gain of 0 Ω, and Offset set to 0 V, versus photon flux.

Fig. 13 shows the TIA output in volts vs. photons detected by the PMT per second for three different P-Gain settings: 0.9 V, 1.0 V, and 1.1 V, while the T-Gain potentiometer was at 0 Ω, and no Offset voltage was applied. The T-Gain is expressed in Ohms (the resistance setting of the potentiometer or RG3), since the measured voltage at test point TP_T-Gain also depends on the Offset. As evident in Fig. 13, increasing the P-Gain increases the slope and hence the sensitivity of the PMT. For low-light sensing applications, the user might want to use the highest P-Gain permitted by the PMT for increased sensitivity, but this also limits the measurement range. In this configuration, the linear range for a P-Gain of 1.1 V is limited to 3x10^7^ photons per second before the TIA signal reaches saturation (black squares, Fig. 13). This is on the order of the abovementioned USB photon counting head (2x10^7^ photons per second. But if now an even wider detection range is required, the P-Gain can be lowered, thereby decreasing the sensitivity but dramatically increasing the measurement range, as demonstrated for P-Gains at 1.0 V and 0.9 V. Here, only measurements up to 7x10^7^ photons per second are presented, but this can linearly be extended until either the PMT or the TIA reaches saturation.

**Fig. 14:**
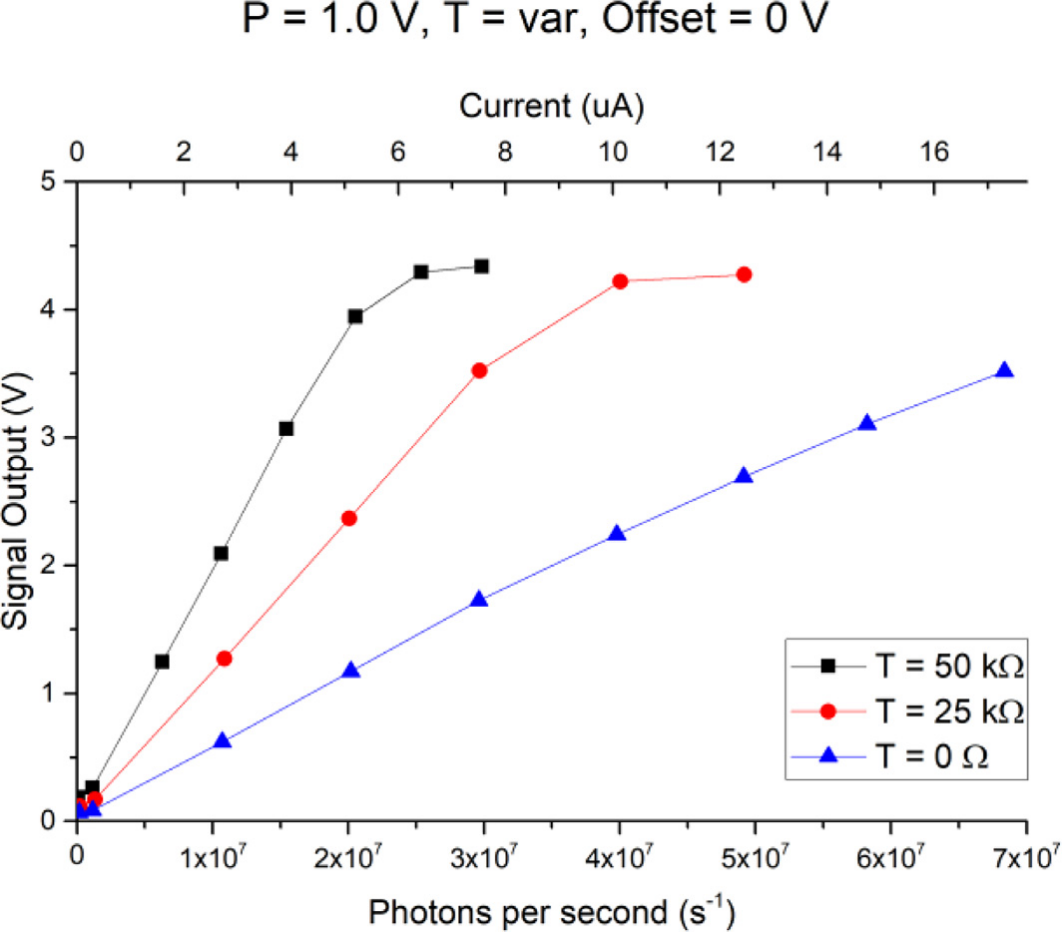
Output signal for varying resistances for the T-gain (0 Ω, 25 kΩ, 50 kΩ), with fixed P-gain setting of 1.0 V, and fixed DC offset of 0 V, versus photon flux. A secondary x-axis (top) also relates TIA input current in uA to converted voltage output.

While it is favored in many applications to optimize the gain of the stage (in this case, the gain of the PMT), not all potential signal sources for the TIA are equipped with a gain control input, such as pin photodiodes or MEMS. In this case, or to further amplify the PMT signal, the TIA has an internal, variable T-Gain. Fig. 14 is essentially the same measurement as in Fig. 13, but for a fixed P-Gain setting of 1.0 V, again zero Offset, but three different potentiometer settings for the T-Gain. Again, this gain allows optimization of sensitivity and measurement range, depending on the application. In this figure, a secondary x-axis (on top) relates the TIA input current to the output voltage. The current was not directly measured but extrapolated using Eqs. (1), (3).

**Fig. 15:**
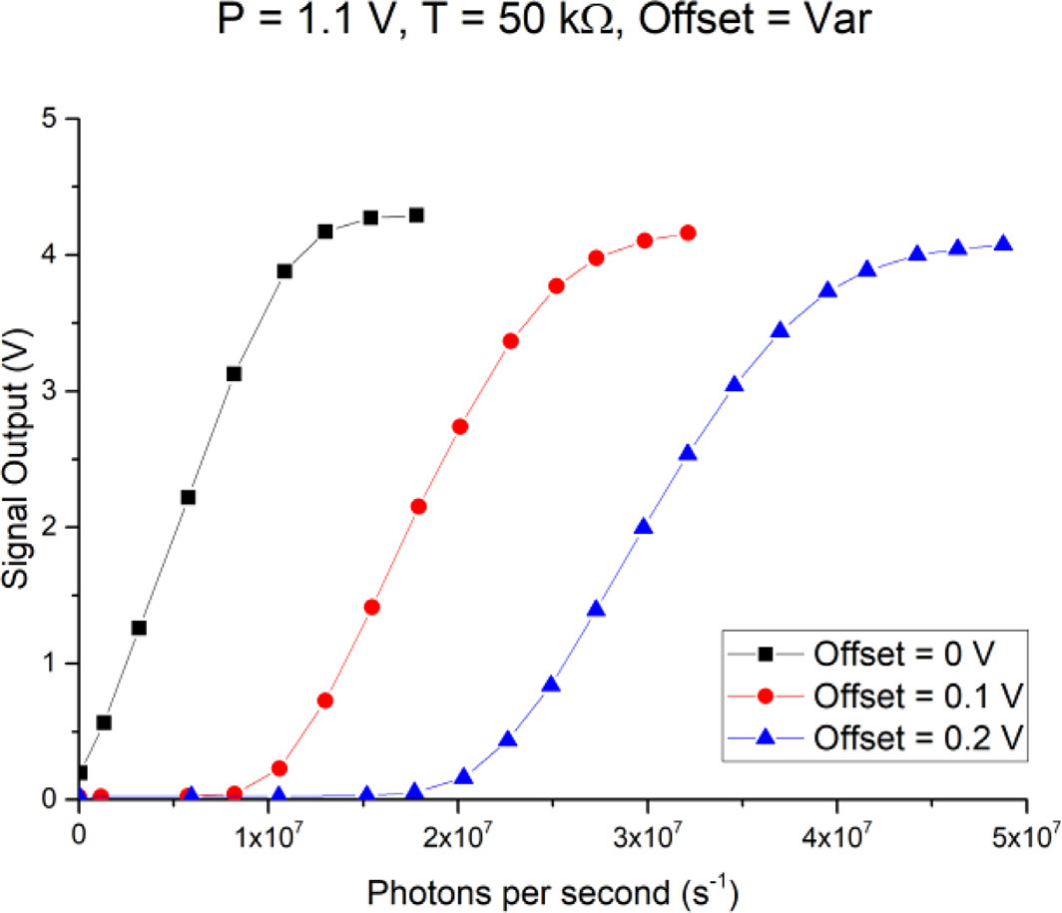
Signal output for varying DC offsets (0 V, 0.1 V, and 0.2 V) with fixed P-gain setting of 1.1 V and fixed resistance for the T-gain of 50 kΩ.

Both previous examples allow for very high sensitivity but hence reach saturation at relatively low current or photon count. In some cases, though, a high sensitivity at an already elevated DC offset is required, for example, when a small optical signal needs to be acquired at maximum sensitivity, but the measurement setup has a high optical background. Another scenario where this is desirable is if small current changes want to be measured on top of a DC current. In this case, the Offset of the TIA can be changed to specifically tune into the area of interest and optimize SNR. In Fig. 15, three curves for fixed P- and T-Gain settings of 1.1 V, and 50 kΩ, respectively, and three different Offset voltages of 0 V, 0.1 V, and 0.2 V are plotted. One example could be where the application requires measurement of small changes in light intensity, but exhibits a background signal of 3 × 10^7^ photons per second. In such a case, this could only be achieved by lowering the P- or the T-Gain if there was no means to compensate for this DC offset. For the commercial USB PMT, this would not be possible, as its linear range is limited to 2x10^7^ photons per second. Now, by compensating for this background signal with the Offset voltage, the measurement range can be shifted, with almost no change in the slope or sensitivity of the TIA. In the above example, with a background signal of 3x10^7^ photons per second, an Offset voltage of around 0.2 V would be ideal, as it allows for measurements under these conditions at maximum sensitivity. If the linear range or sensitivity needs to be changed, both P-Gain and/or T-Gain can also be adjusted in combination with a DC offset. Here, only a few permutations were presented, but the variable resistors allow for an essentially infinite number of gain and offset permutations, enabling optimization for a wide range of different applications.

No error bars are plotted for Fig. 13, Fig. 14, Fig. 15, as they would have been too small to be visible. The average standard deviation per plotted data point was 6 mV, or in the order of 170 photons per second for the highest sensitivity setting of P = 1.1 V, T = 50 kΩ, and Offset = 0 V.

## Declaration of Competing Interest

The authors declare that they have no known competing financial interests or personal relationships that could have appeared to influence the work reported in this paper.
